# A Minor Role of Host Fruit on the Parasitic Performance of *Aganaspis daci* (Hymenoptera: Figitidae) on Medfly Larvae

**DOI:** 10.3390/insects12040345

**Published:** 2021-04-13

**Authors:** Luis de Pedro, Ahlem Harbi, José Tormos, Beatriz Sabater-Muñoz, Francisco Beitia

**Affiliations:** 1 Unidad Asociada de Entomología IVIA-CIB CSIC, Centro de Protección Vegetal y Biotecnología, Instituto Valenciano de Investigaciones Agrarias (IVIA), Ctra. Moncada a Náquera km 4.5, 46113 Moncada, Spain; harbi.ahlem@hotmail.fr (A.H.); b.sabater.munyoz@gmail.com (B.S.-M.); beitia_fra@gva.es (F.B.); 2 Unidad de Zoología, Facultad de Biología, Universidad de Salamanca, 37007 Salamanca, Spain; tormos@usal.es; 3 Department of Crop Protection, Biological Control and Ecosystem Services, Instituto Murciano de Investigación y Desarrollo Agrario y Alimentario, C/Mayor s/n, La Alberca, 30150 Murcia, Spain; 4 High Agronomic Institute of Chott-Mariem, University of Sousse, Chott-Mariem 4042, Tunisia; 5 Smurfit Institute of Genetics, Trinity College, University of Dublin, Dublin2 D02 VF25 Dublin, Ireland; 6 Integrative Systems Biology Group, Institute for Plant Molecular and Cell Biology (IBMCP) from the Spanish National Research Council (CSIC), Polytechnic University of Valencia (UPV), 46022 Valencia, Spain

**Keywords:** host fruit, *Aganaspis daci*, *Ceratitis capitata*, medfly, parasitic performance, olfactory testing, population reduction, host fruit preference, inundative releases, hotspot control

## Abstract

**Simple Summary:**

The medfly, *Ceratitis capitata,* is one of the main pests of citrus and other fruits worldwide. One of the most promising parasitoids for the control of this pest is *Aganaspis daci*, which has been recently discovered in the Mediterranean Basin. The development of fruit pests is strongly affected by the host fruit and this is also expected to affect the parasitic performance of their natural enemies. Therefore, in this study, we measured both the olfactory and parasitic response of female *Aganaspis daci* to different fruit species that can host medfly larvae. This parasitoid was more attracted to apples and uninfested fruit and showed very similar parasitic activity among the different tested fruits. However, the parasitic performance differed significantly depending on the environmental conditions under which the assays were conducted, showing good results in the laboratory and a much poorer performance in greenhouse trials. We conclude that *A. daci* may be a good candidate to control the medfly in a range of different crops, but only when climatic conditions allow normal activity of this species.

**Abstract:**

Host fruit is known to strongly affect the performance of both fruit pests and their potential natural enemies. This is particularly important in the control of tephritid fruit flies, whose larvae develop inside the fruit and thus create a set of foraging problems for parasitoids. In the present study, we assessed the response of female *Aganaspis daci* (Weld) (Hymenoptera: Figitidae)*,* one of the most promising parasitoids for tephritid biocontrol in the Mediterranean Basin, to different potential host fruit species. We measured the olfactory response to medfly-infested and uninfested fruits, and several biological parameters of *A. daci* when different infested fruits were offered under both laboratory and greenhouse conditions. Our results showed that this parasitoid was significantly more attracted to apples and uninfested fruit. Moreover, parasitic activity was similar among the tested fruits under both conditions, showing very high values in the laboratory and a much poorer performance when conditions were variable. This suggests that *A. daci* may be a good candidate to be included in mass releases against the medfly regardless of the affected crop, but only when climate conditions are not expected to hinder its normal activity.

## 1. Introduction

The Mediterranean fruit fly (medfly) *Ceratitis capitata* (Wiedemann) (Diptera: Tephritidae) is currently one of the main pests for citrus and other fruit worldwide. *Ceratitis capitata* is a multivoltine species, is able to feed and develop in more than 330 plant host species, and has adapted to a wide range of climates [1,2]. These reasons have led to the expansion of this species throughout most temperate regions, where it causes significant economic losses due to direct damage to fruit and reduces exports to medfly-free areas [3,4]. Control programs against *C. capitata* have therefore become essential. In this regard, and after many years relying in the use of pesticides [5], the most used control strategies today are environmentally friendly methods such as the sterile insect technique (SIT), mass trapping, chemosterilant traps, or biological control through the use of parasitoids [6,7,8].

One of the most promising parasitoids for medfly control is *Aganaspis daci* (Weld) (Hymenoptera: Figitidae). It is a solitary larval-pupal endoparasitoid of tephritids that was first recorded in Malaysia and Taiwan as a parasitoid of *Bactrocera dorsalis* (Hendel) [9]. Due to the efficiency shown on different hosts from the genus *Bactrocera* Macquart (formerly *Dacus*) in its native area, this species was introduced into different countries for the control of various tephritid pests in the past decades, showing a remarkable biocontrol potential [10,11,12,13,14,15]. These introductions included several releases, mainly in the seventies, which aimed to control natural medfly populations in areas such as mainland France and Reunion Island. However, in these cases, the results obtained were not conclusive [16,17]. The understanding on the relationship between *A. daci* and *C. capitata* changed dramatically in 2003 when, for the first time, this parasitoid was found emerging naturally from medfly pupae, in fig fruits in the Greek island of Chios [18]. Since then, *A. daci* has also been observed attacking medfly in Spain in fig and citrus fruits [19,20], in Syria in guava, grapefruit, loquat, and peach [21,22] and in Tunisia and Morocco also in citrus (F. Beitia, personal communication). These reports suggest that *A. daci* may have adapted to the Mediterranean climate. As such, the parasitoid has established stable populations, where it uses the medfly as a host, in some Mediterranean countries.

Despite its presence in the Mediterranean Basin, the effectiveness of *A. daci* in controlling medfly in this area is still under study. Field recoveries in Greece [18] and Spain [19,20] showed high rates of parasitism, but further observations revealed that the fertility of this species on *C. capitata* was very low compared with other parasitoids in Mediterranean areas [23,24,25,26,27]. Later studies conducted in the Valencian Community (Spain) [28,29] concluded that the low fertility in the field was attributed to the deleterious effect of temperature extremes on *A. daci* adults and juveniles. Despite this, *A. daci* could still oviposit throughout the whole year, causing high mortality of juvenile medfly and successfully reduced their populations. The Mediterranean climate, therefore, does not seem to be optimal for the establishment of several consecutive generations of *A. daci.* However, this species could be considered a promising candidate to be used in inundative releases or in hotspot control against *C. capitata* in the Mediterranean Basin.

The biocontrol potential of a natural enemy may vary substantially depending on the plant species being exploited by its polyphagous host [30]. Hence, the knowledge of how a natural enemy responds to the host plant exploited by the target pest is essential when planning biocontrol programs. This is particularly interesting in the relationship between tephritid fruit flies and their parasitoids. Tephritid pests usually exploit an extraordinary range of host species and the larvae are strongly affected by the properties of the fruit in which they develop [31]. On the other hand, their parasitoids need to oviposit on preimaginal stages of the flies, which cannot be seen from outside the fruit. These parasitoids must efficiently use a variety of visual and, especially, tactile and fruit-derived chemical cues to successfully locate their hosts [32]. The ability of parasitoids to locate infested fruits, detect host larvae inside, and to bore the fruit’s skin to lay their eggs is therefore crucial in the performance as biocontrol agents. However, to date, the host locating ability of *A. daci* on medfly larvae and the effect of host fruits on the foraging processes remains understudied.

Therefore, in the present study, we aimed to assess how the females of *A. daci* respond to the fruits of potential medfly host species from the Mediterranean Basin. We tested the attractiveness of different fruits via olfactory testing. Furthermore, we assessed the effect of these fruits on several biological parameters that are indicative of the parasitic potential and compared these parameters among fruit types to establish a host fruit preference for *A. daci* females. This is expected to be useful to put into perspective the relevance of host fruit in *A. daci* parasitic performance and, as a result, to be able to adequately plan hotspot control programs or inundative releases of *A. daci* against the medfly in the studied area.

## 2. Materials and Methods

### 2.1. Study Centre, Insect Rearing, and Host Fruits

All experiments in this study were performed in compliance with current Spanish law. Insects were obtained from laboratory colonies at the Instituto Valenciano de Investigaciones Agrarias (IVIA, Valencia, Spain). The *A. daci* colony was established in 2010 from individuals reared from field collected medfly larvae in figs from the nearby area of Bétera (Valencia, Spain). Since then, the laboratory rearing has been maintained, using *C. capitata* as a host. Specifically, medfly larvae mixed with an artificial diet mainly constituted by wheat bran, sugar, and brewer’s yeast were offered to parasitoids to allow oviposition, as indicated in de Pedro [33] (rearing conditions 27 ± 2 °C, 65% ± 10% RH, 16:8 (L:D) photoperiod). The medfly has been reared at the IVIA since 2008, using the method of Pérez-Hinarejos and Beitia [25] (rearing conditions: 27 ± 2 °C, 65% ± 10% RH, 16:8 (L:D) photoperiod), in which medfly eggs were sown on the same type of the artificial diet mentioned above. The fruits used in the study were chosen based on several aspects such as their organoleptic properties, their availability in the market and their socioeconomic relevance in the Mediterranean area. Four types of fruits were selected: apple (*Malus domestica* Borkh, cv. Royal Gala), orange (*Citrus sinensis* (L.) Osbeck, var. Navel), peach (*Prunus persica* L., var. Nectarin), and clementine mandarins (*Citrus clementina* Ex. Hort. Tan., var. Clemenules). All these fruits were provided by local organic farmers. At IVIA, fruits were washed with chlorinated tap water and stocked in a cold-storage room at 8 ± 1 °C and 50% ± 5% RH until their use for trials. Due to their availability throughout the year, apples were used as controls, i.e., the reference fruit for comparisons between treatments. 

### 2.2. Experimental Design

Three experiments were carried out to assess the response of *A. daci* to the abovementioned fruit species.

#### 2.2.1. Olfactory Testing

The response of *A. daci* to different olfactory stimuli was assessed by using a Y-tube olfactometer (Analytical Research Systems, ARS lt, Gainesville, FL, USA) in a series of trials performed under controlled conditions (23 ± 2 °C, 60% ± 10% RH, 2516 lux). The glass Y-tube was 2.4 cm of diameter, with a 13.5 cm base and two arms, each 5.75 cm in length. This was connected to an air pump producing a unidirectional airflow of 150 mL/min from the arms to the base (wind speed of 0.005 m/s). Airflow was established based on previous experiments with similar-sized insects [30,34,35,36]. The air pump was connected to two 5-L crystal jars containing the odor sources to be evaluated (in this case, test fruits). Each odor source was then connected to one of the arms of the Y-tube olfactometer. This methodology has been already described in previous works [36,37].

For all olfactory tests, 8-day-old *A. daci* females with previous parasitic experience on medfly larvae (i.e., obtained from the laboratory colony) were used. These specimens were individually isolated in 10-mL plastic tubes and left for 2–3 h in the olfactometer room to adapt to the assay conditions. In a first set of trials, our aim was to compare the response of *A. daci* females to infested vs. uninfested fruits, using apples, oranges, and peaches. Furthermore, we assessed whether time since infestation affected the female response, by comparing the attractiveness of uninfested apples vs. 1-day-old and 4-day-old infested apples. Finally, we also assessed the olfactory response to different types of fruits, all of which had the same infestation age. One-day-old infested apples, peaches, and oranges were pair-wise compared in these trials. Fruit infestation was artificially performed according to Martins et al. [38], digging 10 holes (5 mm diameter, 10–15 mm depth) in each fruit with a puncher and placing three medfly larvae (late L2—early L3 stages) in each hole. Holes were subsequently closed with its corresponding fruit plug. Artificially infested fruits were stored at 25 ± 2 °C in protected cages to avoid accidental infestation, for 1–4 days depending on the trial. Uninfested control fruits were subjected to the same drilling and storage process and thus reached the same ripeness stage, but no larvae were introduced. 

*Aganaspis daci* females were tested individually in olfactory trials. Each female was placed at the base of the Y-tube using a small brush and allowed 15 min to respond [39]. A response was considered “positive” if the female walked at least 3 cm into one of the arms within this 15 min period, and was scored according to the chosen arm/odor source. All other responses were considered “negative”, and the female was classified as a “non-responder” and discarded from subsequent analyses. This procedure was repeated until at least 30 “positive” responses were recorded for each paired combination. To minimize any uncontrolled effect on the choice of the parasitoids, every 5 females/tests the Y-tube arms were flipped, and every 10 females/tests the Y-tube and the jars were thoroughly rinsed with soap, water, and acetone and then air-dried. 

#### 2.2.2. Laboratory Assays

The laboratory trials were performed in a climatic chamber under controlled conditions (25 ± 2 °C; 70% ± 10% RH; 16:8 (L:D) photoperiod). Plastic boxes (30 cm × 25 cm × 20 cm), each with a muslin window for ventilation on the upper surface and containing water and sugar ad libitum, were used as parasitism units. Three 6–8-day-old *A. daci* mating couples were placed inside each parasitism unit. Artificially-infested fruits (as mentioned above according to [38] with 30 late L2—early L3 medfly larvae per fruit) were deposited inside parasitism units under two distinct treatments, establishing two different subassays as follows: “no-choice” tests in which three infested fruit of only one species were deposited in the parasitism units, or “host-choice” tests in which six infested fruits of two different species (3 + 3) were introduced. In both tests, each infested piece of fruit was isolated in a 200-mL plastic cup containing a layer of vermiculite to provide the larvae with a suitable substratum to pupate. The four abovementioned fruit species (apple, orange, peach, and clementine) were used in both tests, with apples always as the reference fruit in the host-choice tests. Three replicates of laboratory trials were performed, each consisting of fifteen experimental units: five of each fruit (no-choice) and five of fruit combination (host-choice). Four additional parasitism units (two per treatment), containing no parasitoids, were used in each replicate to assess the natural mortality of the medfly larvae. The infested fruits were exposed to the parasitoids for 4 days; then, medfly pupae were recovered and put in ventilated Petri dishes (one per unit and fruit type) that were kept in a climatic chamber, as above, until the emergence of parasitoid and medfly adults. Emergences and closed puparia were counted. From these data, we analyzed the following variables to assess host preference by *A. daci*: effective fertility (as the number of descendants produced by the parasitoids), parasitoidism (=parasitoidism rate, as the number of descendants per recovered pupae), induced mortality (=mortality rate, as the number of pupae that remain closed per recovered pupae due to parasitoid activity), population reduction (as the sum of induced mortality plus parasitoidism), and offspring sex ratio (proportion of *A. daci* females among the total number of emerged parasitoids, *n* = ♀♀/♂♂ + ♀♀).

#### 2.2.3. Semi-Field Assays

Semi-field trials were conducted to simulate natural conditions inside a greenhouse at IVIA, between March and July of 2014. The greenhouse was equipped with four plastic-framed mesh cages (125 cm × 120 cm × 70 cm), each constituting a parasitism unit and containing water, sugar and honey as nutritional sources. Two translucent plastic top-open boxes (40 cm × 40 cm × 40 cm) were placed inside each of the cages, each box containing nine medfly-infested fruits deposited on a thin layer of vermiculite as described above. The fruit species differed between the two boxes in each cage, thus establishing a “host-choice” test. Three 6–8-day-old *A. daci* mating couples were released inside the four cages where the females could freely forage on the infested fruits for 4 days, and apples were again used as the reference fruit and compared with another fruit (orange, peach, or clementine) in each trial. After 4-days of exposure, all plastic boxes were closed and taken to the laboratory where the medfly pupae were recovered, put in ventilated Petri dishes (one per unit and fruit type) and kept under controlled conditions (25 ± 2 °C; 70% ± 10% RH; 16:8 (L:D) photoperiod) until adults emerged. Emergences and closed puparia were counted, as indicated for the laboratory trials. Each pair-wise host-choice test was replicated three times. In each replicate, three infested fruits of each type were placed out of the cages, as control treatments to assess natural medfly larvae mortality. Effective fertility, parasitoidism, induced mortality, population reduction, and offspring sex ratio were analyzed as for laboratory trials.

### 2.3. Data Analysis

Data were analyzed using SPSS v22.0 (IBM SPSS, Chicago, IL, USA) with a significance set at *p* = 0.05. Prior to the analysis, data normality was checked and data were transformed, where necessary.

Choice data of *Aganaspis daci* females derived from Y-tube olfactometer tests were analyzed with a Pearson’s chi-squared (χ^2^) test to compare the attractiveness between each pair of odor sources. 

For laboratory and semi-field assays, Pearson’s chi-squared (χ^2^) test was also used to determine any significant differences in the offspring sex ratio depending on the type of fruit exposed. Linear mixed-model ANOVAs, with one fixed factor (fruit) and one random factor (replicate), were performed to test the effective fertility, parasitoidism, induced mortality and population reduction displayed by *A. daci* depending on the host fruit, in the no-choice laboratory tests. In the choice tests, the same analysis was performed but with the random factor (“replicates” in the laboratory assays, and “mesh cages” in the semi-field assays) hierarchical to fruits, since the two kinds of fruits were together in the same place. Induced mortality (i.e., corrected mortality) was calculated using the Schneider–Orelli formula [40] as follows:

Induced mortality (%) = [(Treatment mortality % − Control mortality %)/(100 − Control mortality)] × 100

Here, population reduction is defined as the sum of induced mortality plus parasitoidism.

## 3. Results

### 3.1. Olfactory Testing

**Figure 1:**
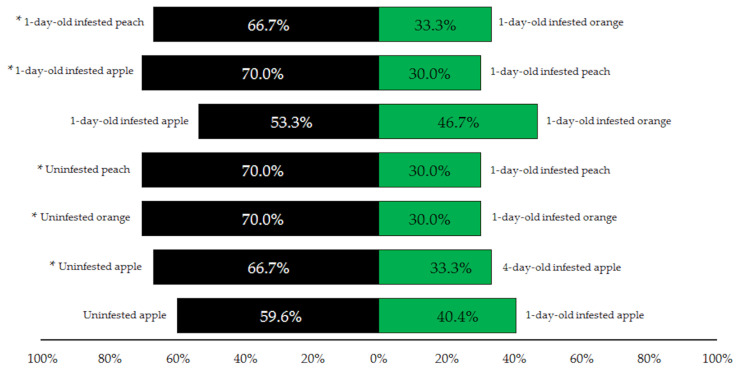
Percentages of *A. daci* females responding to each odor source (host fruit) in the different paired combinations of the olfactory tests. Asterisk (*) indicates significant differences (*p* ≤ 0.05).

Olfactory tests revealed that *A. daci* females were more attracted to uninfested fruit than medfly-infested fruit (Figure 1). This preference was statistically significant when uninfested fruit was compared with 1-day-old infested peaches and oranges (χ^2^ = 9.60, df = 1, *p* = 0.004), and with 4-day-old infested apples (χ^2^ = 6.67, df = 1, *p* = 0.019). When comparing uninfested apples with 1-day-old infested apples, higher attraction to uninfested fruits was still observed, but it was not statistically significant (χ^2^ = 4.25, df = 1, *p* = 0.061). Pair-wise comparisons among different fruit types revealed *A. daci* females had a preference for apple odors, which was significant when compared with peach (χ^2^ = 9.60, df = 1, *p* = 0.004) but not significant when compared with orange (χ^2^ = 0.27, df = 1, *p* = 0.797). Paradoxically, when comparing orange to peach, there was significant preference for peach odor (χ^2^ = 6.67, df = 1, *p* = 0.019). These results suggest that *A. daci* females, based on olfactory stimuli, seem to be more attracted by apple than by the two other fruits, and that peach is preferred to orange.

### 3.2. Laboratory Assays

Contrary to what was expected based on the olfactory responses, laboratory assays did not reveal a preference of *A. daci* females towards infested apples, oranges, clementines, or peaches, but it must be considered that the attraction towards volatiles (results in olfactometer) is completely different to the feasibility to parasitize (results in laboratory assays). In no-choice tests, ANOVA did not show significant differences among apples and any other fruits for parasitoidism, effective fertility, induced mortality, and population reduction (Table 1 and Table 2), with a few exceptions. In this regard, effective fertility was significantly higher in *A. daci* parasitizing apple when compared with orange (*p* = 0.043), and the population reduction caused by parasitoids on apple was also significantly higher than on peach (*p* = 0.006) (Table 1 and Table 2). Meanwhile, chi-squared test revealed that the offspring sex ratio was significantly different between apple and clementine (χ^2^ = 5.28, df = 1, *p* = 0.022), with a higher proportion of females in apple; and between apple and orange (χ^2^ = 27.21, df = 1, *p* < 0.001), with higher values in the latter (Table 2). However, no significant differences were observed between apple and peach (χ^2^ = 0.09, df = 1, *p* = 0.768). Additionally, for each fruit, this test also revealed a biased sex ratio in the offspring obtained from apple (χ^2^ = 78.92, df = 1, *p* < 0.001), clementine (χ^2^ = 43.62, df = 1, *p* < 0.001), peach (χ^2^ = 28.50, df = 1, *p* < 0.001), and orange (χ^2^ = 3.52, df = 1, *p* = 0.03). In all cases, the sex ratio was female-biased.

In host-choice tests, ANOVA revealed no significant differences among apples and any of the other fruits for the measured variables (Table 1 and Table 3). As in no-choice tests, both parasitoidism and effective fertility were numerically higher in apple than peach and orange (Table 3), but these differences were not statistically significant (Table 1). Similarly, population reduction was also higher in apple than in peach (Table 3), but statistical analysis did not support this difference (Table 1). Regarding the offspring sex ratio, chi-squared test revealed significant differences between apple and clementine (χ^2^ = 13.45, df = 1, *p* < 0.001) and apple and peach (χ^2^ = 7.69, df = 1, *p* = 0.006) (in both cases with a lower proportion of females emerged from apples) but not between apple and orange (χ^2^ = 0.31, df = 1, *p* = 0.582). In each fruit, offspring were significantly females biased (apple vs. clementine: χ^2^ = 40.95, df = 1, *p* < 0.001; clementine: χ^2^ = 109.48, df = 1, *p* < 0.001; apple vs. peach: χ^2^ = 52.68, df = 1, *p* < 0.001; peach: χ^2^ = 52.29, df = 1, *p* < 0.001; apple vs. orange: χ^2^ = 16.70, df = 1, *p* < 0.001; orange: χ^2^ = 8.31, df = 1, *p* = 0.005).

In summary, the abovementioned results indicate that *A. daci* parasitic activity in apple and clementine was very similar, showing high effective fertility values and rates of parasitoidism. Both parameters were numerically lower in orange and even more so in peach, but the higher induced mortality observed in these fruits led to similar overall values of population reduction. Considering apples as a reference and population reduction as the most complete indicator of parasitoid activity, the host fruit preference under laboratory conditions, which was very weak, could be established as similar between apple and clementine and, as in the olfactometer test, lower for orange and peach.

### 3.3. Semi-Field Assays

The results obtained under greenhouse conditions were similar to those observed in the laboratory. Semi-field assays did not show a clear preference of the parasitoid to apples, oranges, clementines, or peaches. ANOVA did not reveal significant differences among apples and the other fruits for parasitoidism, effective fertility, induced mortality, and population reduction (Table 4 and Table 5). All these variables were very similar between apple and orange, while the comparisons “apple vs. clementine” and “apple vs. peach” showed higher (but not statistically significant) values of parasitoidism, population reduction, and, especially, effective fertility for apples (Table 5). Based on these results and especially on population reduction values, a very weak host fruit preference was observed for apple compared to the other tested fruits, similar than in the laboratory assays.

Pearson’s chi-squared test only revealed significant differences between the offspring sex ratio of apples and peaches (χ^2^ = 4.31, df = 1, *p* = 0.043), but not in the comparisons “apple vs. clementine” (χ^2^ = 1.84, df = 1, *p* = 0.184) nor “apple vs. orange” (χ^2^ = 0.53, df = 1, *p* = 0.499). Additionally, in each fruit, a significantly female-biased offspring was revealed (apple vs. clementine: χ^2^ = 56.70, df = 1, *p* < 0.001; clementine: χ^2^ = 7.51, df = 1, *p* = 0.010; apple vs. peach: χ^2^ = 72.71, df = 1, *p* < 0.001; peach: χ^2^ = 35.49, df = 1, *p* < 0.001; apple vs. orange: χ^2^ = 62.21, df = 1, *p* < 0.001; orange: χ^2^ = 70.51, df = 1, *p* < 0.001).

## 4. Discussion

The plant species that hosts an insect pest is known to be a key factor affecting the predatory or parasitic ability of its natural enemies [30,41,42]. This effect is particularly interesting in the case of the medfly *Ceratitis capitata*, one of the most economically damaging fruit pests in the Mediterranean Basin, due to the enormous range of potential hosts [1,2]. Therefore, in the present work, we evaluated the performance of the larval–pupal parasitoid *Aganaspis daci*, one of the most promising parasitoids of the medfly, on several fruit types that are economically relevant in the Mediterranean Basin and constitute potential hosts for medfly larvae. The first parameter considered in this study was the olfactory attractiveness of these fruits to the females of *A. daci*. Olfactory cues are essential for the orientation and parasitic behavior of parasitoids, since they are known to respond to allelochemicals released by plants and/or host insects as source of information for habitat location and host selection [43,44,45,46]. Interestingly, in the present study *A. daci* females were more attracted to uninfested fruit than to medfly-infested fruit and, moreover, in the case of apples, this innate preference for uninfested fruit was even more marked when compared to the 4-day-old infested fruits. This trend is completely opposite to that observed for other fruit fly parasitoids, such as the close species *Aganaspis pelleranoi* (Brèthes) [47] and the braconids *Doryctobracon aureolatus* (Szépligeti) [48] or *Diachasmimorpha longicaudata* (Ashmead). *Aganaspis pelleranoi* has been reported to be more attracted to volatiles of guavas infested with frugivorous larvae [47]. For its part, the females of *D. longicaudata*, which is one of the most widely employed species against tephritids [13], have also shown a significant preference for fruits infested with larvae of the medfly and/or other fruit flies [36,48]. In this regard, larvae in rotting fruits have long been believed to release or induce the formation of volatiles that are attractive to parasitoids when compared with uninfested ripe fruit [49,50,51,52]. For example, para-ethylacetophenone, an analog of a tephritid parasitoid attractant, has been identified as a major constituent of the volatiles produced by several species of tephritids including the medfly, and has been found to be attractive to *D. longicaudata* females [31]. According to our results, these volatiles seem to be innocuous or even repellent for *A. daci*, probably due to any of the differences observed among the sensillar equipment of this species and that of other medfly parasitoids including *D. longicaudata* and *A. pelleranoi* [53,54,55,56]. Thus, host location in *A. daci* seems to be initially driven by an attractive effect of the volatiles emitted exclusively by rotting fruits. Once the fruit surface is reached, we hypothesize that females may locate the host mainly by vibrotaxis using their mechanoreceptive sensillae, which are known to be specially advanced among those of fruit fly parasitoids [55]. Moreover, vibrotaxis has also been reported to allow close-range location in *A. pelleranoi* [47].

Olfactory pair-wised comparisons among fruit types revealed that this parasitoid seems to be more attracted to apples, followed by peaches and oranges. This could be explained by an attractive effect of some of the volatiles released by ripe apples, but also by a repellent effect of some compound emitted by the other fruits. In this regard, para-ethylacetophenone is known to be also a component of the volatiles produced by oranges [57], thus reinforcing the hypothesis that this compound may be repellent to *A. daci* females. Contrarily to our results, similar previous studies showed that *D. longicaudata* females were significantly more attracted to peach and citrus than apples, both using uninfested [30] and medfly-infested [36] fruits. All these trends highlight the specificity of volatile responses among fruit fly parasitoids and emphasize the need to perform further experiments to determine the specific compounds involved in olfactory preferences.

Laboratory and greenhouse trials confirmed that *A. daci* could parasitize tephritid larvae on different host fruits, supporting previous observations. This species had been previously observed parasitizing larvae from the genus *Bactrocera* Macquart on mango and cempedak in its area of origin [9], on guava in Egypt [15], and even on olive fruits in laboratory trials in Greece [58]. Moreover, *A. daci* has been reported emerging from larvae of *C. capitata* on figs [18,20], citrus [20], apples [59,60], loquats, guavas, grapefruits, and peaches [21,22]. 

These trials also revealed very similar parasitic performance of *A. daci* across the different host fruits, in spite of the observed olfactory preferences. In laboratory trials, choice tests did not reveal significant differences in any of the pair-wise comparisons, while no-choice tests only revealed significantly higher effective fertility in apples when compared with orange and greater population reduction ability on apple compared with peach. All these parameters tended to be higher in apple and clementine than in peach and orange, with the exception of induced mortality. This reveals a slight preference for the apple and clementine, which was in line with olfactory tests and suggests a certain association between preference and parasitoidism rate. This has been studied in other fruit fly parasitoids with conflicting results [30,49,61]. However, the experimental design of laboratory trials, in which little space is available for parasitoids and the access to the fruit surface is easy, leads to the belief that other factors beyond olfactory stimuli may better explain the observed results. Among these factors, several intrinsic characteristics of the fruits are known to have a decisive influence on the host–parasitoid interactions and, particularly, on host accessibility [62]. Fruit size and pulp depth, which are usually correlated, are often considered as the most important factors, since larvae sheltered deep within fruit pulp are more difficult to detect and less accessible to parasitoids [63]. Moreover, the development and movement of frugivorous larvae is influenced by fruit texture, mainly due to the physical features of the pulp and the consequent intrinsic forces [64,65,66,67]. The fruit skin firmness has a major influence on the ability of parasitoids to find the host and bore the peel to lay their eggs, this being easier in fruits of thin and soft skin, which offer low penetrating resistance [62,63,68]. Our results, in which mean population reduction was above 70% in all cases, suggest that the physical properties of all the fruits tested are suitable for high parasitic activity of *A. daci* on the medfly. This had already been proved for peaches [22] and apples [59,60], on which *A. daci* had already shown higher parasitoidism rates than *D. longicaudata* when released together [60]. This was contrary to expectations considering the longer ovipositor of this braconid [9,69,70], the hypothesis of Sivinski [71] that parasitoids with short ovipositor can only infest the larvae found in small fruits and the high firmness of the mesocarp in apples [65]. As in de Pedro et al. [60], we should conclude that the advanced sensillar equipment of *A. daci* [55] makes this parasitoid very efficient at searching for hosts inside fruit, most probably allowing a better detection of larvae even in fruits of significant size.

Greenhouse trials were designed to imitate conditions in the field. Unlike in laboratory trials, in semi-field assays olfactory and visual cues are expected to play an essential role in fruit/host searching and location by parasitoids [32], due to the greater space available and the large distance at which these cues are perceived [72]. Results showed, again, no significant differences among fruits in *A. daci* parasitic performance, even though we should highlight the numerally higher effective fertility and parasitoidism in apple and orange and the substantial induced mortality observed in peach. However, the values obtained in these trials for all fruits were surprisingly low compared with previous studies with the same experimental design [59,60]. This poor performance cannot be attributed to the host fruit species or to intrinsic factors of parasitoids, as previous studies have used apples and individuals of the same age and with the same parasitic experience as those used in this work. Considering the extreme sensitivity of *A. daci* to climatic conditions and especially to temperature [28,29], together with the high temperatures recorded throughout the greenhouse trials (with an average over 27 °C in most weeks of study), we hypothesized that this and some other environmental factors may have negatively affected its development and/or parasitic performance. In this regard, temperature, wind speed and direction, humidity, or light intensity are known to affect the olfactory response of parasitoids to fruits or host odors and, consequently, the host location process [43,73,74]. 

Temperature is also considered as the main factor affecting another of the measured parameters in our study, the sex ratio. A previous work [28] stated that, in the suitable developmental temperature range, the proportion of female offspring rises with increasing temperatures. In our study, offspring were female-biased in every trial and fruit species, and even more strongly under greenhouse conditions, where high temperatures were undoubtedly reached in the period of study. Female bias appears to be very common among hymenopteran parasitoids [75] and is always a desirable feature in biocontrol agents since females exert parasitic activity. However, this is not as relevant in inundative releases or hotspot control as it is in inoculative releases, where most control is provided by offspring of released organisms [76].

The host fruit preference suitability for *A. daci* females was established under the different conditions considered in this work. In laboratory and semi-field trials, this preference was established based on population reduction, which best summarizes the deleterious effect produced by parasitoids on pest populations. This innate preference was not strong in any case, in line with the observations of Ali [22] in some preliminary trials conducted in Western Syria and as one would expect for a parasitoid whose hosts are so polyphagous. In other medfly parasitoids, namely *D. longicaudata*, a strong hierarchical preference among apple, fig, orange, and peach has been reported by Segura et al. [30], but it should be addressed that, in this case, fruits were uninfested. Indeed, this and other species [77,78,79] are known to no longer respond according to innate preferences when hosts are present, seeking instead for those habitats in which the probability of host encounter is higher.

In our study, preference tended to be slightly biased towards apples, which are not amongst the most favorable hosts for *C. capitata* [66], unlike stone fruits and citrus, whose economic relevance in Spanish Mediterranean areas is much higher [80]. This could put the success of *A. daci* releases in this area into question. However, as mentioned before, the parasitic performance observed on stone fruits and citrus was not statistically lower, with very good results under controlled conditions and a weaker performance in a more varying environment. This information underlines climatic conditions, better than host fruit, as the key conditioning factor affecting the performance of *A. daci* as a biocontrol agent against the medfly in the Mediterranean Basin.

## 5. Conclusions

The present research constitutes, to our knowledge, the first exhaustive study on the effect of host fruit on the parasitic performance of *A. daci* against medfly, following preliminary results provided by Ali [22] in Western Syria. On the one hand, we conducted the first olfactometer trials reported for this parasitoid species, in which a clear preference for uninfested fruit over medfly-infested fruit was observed. One explanation to this fact may be a certain repellent effect caused by some volatile emitted by medfly larvae. Considering the observed preference for apples and peaches over oranges, and the presence of para-ethylacetophenone as a major constituent of the volatiles produced by both oranges and the medfly [31,57], this compound may be suggested as a possible repellent agent to *A. daci* females. On the other hand, we also confirmed, via laboratory and greenhouse trials, that *A. daci* is able to efficiently parasitize medfly larvae on different fruit species that are economically relevant in the Spanish Mediterranean area. This parasitic performance did not differ significantly among the host fruits, but between the different conditions under which trials were conducted. Under controlled conditions, the reduction of medfly populations caused by *A. daci* females was above 70% in every tested fruit, suggesting that physical properties of all these fruits are suitable for an adequate activity of this parasitoid. However, in greenhouse trials simulating natural conditions, the parasitic performance was very poor, which, considering similar previous studies [59,60], may be attributed to some environmental factor that could have negatively affected *A. daci* survival, development or parasitic ability. Thus, despite a certain host fruit preference being determined for each type of trial (with a slight dominance of apples in all cases), the effect of host fruit species on the parasitic performance of *A. daci* seems to be less relevant than, for example, that of environmental factors. In conclusion, our findings recommend the use of *A. daci* in biocontrol programs against the medfly in different crops, both in hotspot control or through inundative releases, but only at appropriate times of the year.

## Figures and Tables

**Table 1:** Linear mixed-model ANOVAs comparing parasitoidism, effective fertility, induced mortality and population reduction of *A. daci* between host fruit, in no-choice and host-choice tests, under laboratory conditions. df = degrees of freedom; RV = residual variance; BV = block variance. Asterisks (*) indicate significant differences in each comparison.

Parameter		No-Choice Tests			Host-Choice Tests		
		Apple vs. Clementine	Apple vs. Peach	Apple vs. Orange	Apple vs. Clementine	Apple vs. Peach	Apple vs. Orange
Parasitoidism	F	1.12	2.90	8.14	0.26	8.57	0.06
	df	*1,24*	*1,24*	*1,24*	*1,24*	*1,24*	*1,24*
	*p*	0.402	0.231	0.104	0.662	0.100	0.824
	σ^2^ (RV)	48.77	38.34	57.66	34.23	28.67	37.44
	σ^2^ (BV)	1 × 10^−8^	1 × 10^−7^	1 × 10^−9^	1 × 10^−9^	1 × 10^−9^	1 × 10^−7^
Effective fertility	F	0.01	0.10	21.63	3.50	17.26	16.17
	df	*1,24*	*1,24*	*1,24*	*1,24*	*1,24*	*1,24*
	*p*	0.946	0.783	0.043*	0.202	0.053	0.057
	σ^2^ (RV)	43.55	48.59	32.68	40.33	27.57	31.12
	σ^2^ (BV)	1 × 10^−9^	1 × 10^−8^	1 × 10^−8^	1 × 10^−8^	1 × 10^−9^	1 × 10^−9^
Induced mortality	F	0.04	0.12	2.64	12.54	0.66	0.56
	df	*1,24*	*1,24*	*1,24*	*1,24*	*1,24*	*1,24*
	*p*	0.852	0.765	0.245	0.071	0.502	0.531
	σ^2^ (RV)	39.23	44.54	52.34	28.11	31.55	36.68
	σ^2^ (BV)	1 × 10^−9^	1 × 10^−8^	1 × 10^−8^	1 × 10^−8^	1 × 10^−7^	1 × 10^−8^
Population reduction	F	2.48	177.16	3.62	0.11	0.23	0.02
	df	*1,24*	*1,24*	*1,24*	*1,24*	*1,24*	*1,24*
	*p*	0.256	0.006 *	0.198	0.770	0.678	0.896
	σ^2^ (RV)	37.12	42.68	50.12	41.12	46.89	38.22
	σ^2^ (BV)	1 × 10^−8^	1 × 10^−8^	1 × 10^−8^	1 × 10^−9^	1 × 10^−8^	1 × 10^−9^

**Table 2:** *Aganaspis daci* mean parasitoidism, effective fertility, induced mortality, population reduction (mean ± SE) and offspring sex ratio (*n* = ♀♀/♂♂ + ♀♀) comparing different fruit species in no-choice tests under laboratory conditions. Asterisks (*) indicate significant differences in each comparison.

Parameter	Apple vs. Clementine		Apple vs. Peach		Apple vs. Orange	
	Apple	Clementine	Apple	Peach	Apple	Orange
Parasitoidism (%)	59.5 ± 4.6	54.1 ± 5.5	49.4 ± 5.7	32.5 ± 5.2	59.6 ± 4.1	45.1 ± 5.6
Effective fertility	43.1 ± 3.6	42.5 ± 4.1	37.1 ± 4.7	21. 7 ± 3.8	48.3 ± 4.7 *	35.1 ± 3.6 *
Induced mortality (%)	34.5 ± 4.8	35.8 ± 5.8	49.6 ± 6.3	51.9 ± 7.6	19.8 ± 2.8	37.4 ± 5.5
Population reduction (%)	94.0 ± 1.9	89.8 ± 3.1	99.0 ± 1.2 *	84.4 ± 2.5 *	79.4 ± 4.0	82.4 ± 1.6
Offspring sex ratio	0.74 *	0.68 *	0.70	0.70	0.63 *	0.66 *

**Table 3:** *Aganaspis daci* mean parasitoidism, effective fertility, induced mortality, population reduction (mean ± SE) and offspring sex ratio (*n* = ♀♀/♂♂ + ♀♀) comparing different fruit species in host-choice tests under laboratory conditions. Asterisks (*) indicate significant differences in each comparison.

Parameter	Apple vs. Clementine		Apple vs. Peach		Apple vs. Orange	
	Apple	Clementine	Apple	Peach	Apple	Orange
Parasitoidism (%)	48.5 ± 6.6	50.8 ± 3.5	51.3 ± 4.1	26.0 ± 5.4	60.3 ± 5.8	47.8 ± 5.1
Effective fertility	36.5 ± 4.9	41.9 ± 2.9	35.7 ± 3.0	16.7 ± 3.9	45.5 ± 4.6	31.3 ± 3.3
Induced mortality (%)	27.4 ± 5.6	26.5 ± 3.1	46.0 ± 5.0	44.9 ± 7.4	16.2 ± 2.5	23.8 ± 4.6
Population reduction (%)	75.9 ± 7.4	77.4 ± 4.9	97.4 ± 2.1	70.7 ± 4.1	76.6 ± 5.8	71.6 ± 2.3
Offspring sex ratio	0.69 *	0.77 *	0.71 *	0.81 *	0.61	0.59

**Table 4:** Linear mixed-model ANOVAs comparing parasitoidism, effective fertility, induced mortality, and population reduction of *A. daci* between combinations of host fruit under greenhouse conditions. df = degrees of freedom; RV = residual variance; BV = block variance.

Parameter		Apple vs. Clementine	Apple vs. Peach	Apple vs. Orange
Parasitoidism	F	1.70	1.81	0.11
	df	*1,18*	*1,18*	*1,18*
	*p*	0.311	0.100	0.741
	σ^2^ (RV)	12.14	18.13	14.33
	σ^2^ (BV)	1 × 10^−8^	1 × 10^−8^	1 × 10^−8^
Effective fertility	F	0.13	0.22	0.03
	df	*1,18*	*1,18*	*1,18*
	*p*	0.753	0.686	0.853
	σ^2^ (RV)	23.12	24.66	29.24
	σ^2^ (BV)	1 × 10^−8^	1 × 10^−9^	1 × 10^−8^
Induced mortality	F	0.66	0.61	0.47
	df	*1,18*	*1,18*	*1,18*
	*p*	0.502	0.516	0.565
	σ^2^ (RV)	15.22	23.33	19.22
	σ^2^ (BV)	1 × 10^−9^	1 × 10^−8^	1 × 10^−8^
Population reduction	F	8.99	2.38	2.59
	df	*1,18*	*1,18*	*1,18*
	*p*	0.096	0.263	0.249
	σ^2^ (RV)	18.22	28.44	24.12
	σ^2^ (BV)	1 × 10^−^^8^	1 × 10^−^^8^	1 × 10^−^^8^

**Table 5:** *Aganaspis daci* mean parasitoidism, effective fertility, induced mortality, population reduction (mean ± SE), and offspring sex ratio (*n* = ♀♀/♂♂ + ♀♀) comparing different fruit species in host-choice tests under greenhouse conditions. Asterisks (*) indicate significant differences in each comparison.

Parameter	Apple vs. Clementine		Apple vs. Peach		Apple vs. Orange	
	Apple	Clementine	Apple	Peach	Apple	Orange
Parasitoidism (%)	11.5 ± 4.6	3.0 ± 1.5	16.2 ± 6.1	5.0 ± 2.2	14.5 ± 3.9	13.0 ± 4.7
Effective fertility	27.3 ± 10.8	6.7 ± 3.5	34.7 ± 11.4	9.2 ± 4.2	34.6 ± 9.4	32.6 ± 9.3
Induced mortality (%)	19.3 ± 3.3	18.4 ± 4.2	18.2 ± 4.2	22.6 ± 6.1	9.8 ± 2.7	7.8 ± 1.9
Population reduction (%)	30.7 ± 7.2	21.4 ± 5.2	34.4 ± 7.3	27.6 ± 6.7	24.3 ± 4.4	20.8 ± 2.9
Offspring sex ratio	0.79	0.82	0.78 *	0.87 *	0.77	0.79

## Data Availability

The data presented in this study are available on request from the corresponding author.
